# Supplementary material for: Enhancing Lignocellulose Degradation and Mycotoxin Reduction in Co-Composting with Bacterial Inoculation
Source: Microorganisms. 2025 Mar 18;13(3):677. doi: 10.3390/microorganisms13030677 (PMC11946631; doi:10.3390/microorganisms13030677)
Supplement: Supplementary file 1 [file microorganisms-13-00677-s001.zip › microorganisms-3489438-supplementary.pdf]

Supplementary material for

Effects of bacterial agents on aerobic co-composting of mycotoxins-contaminated silage and rape straw

Figure caption

Figure S1 Bacterial community composition at (a) phylum and (b) genus level during compost at day 1, 30 and 60, inoculated without (CK) or with *Bacillus subtilis* (B), *Paenibacillus* sp. (P), *Weissella paramesenteroides* (WSP) and their combinations (B+P, BP; WSP+B, WSPB; WSP+P, WSPP; and P+B+WSP, WSPBP) (relative abundance less than 1% is classified as others).

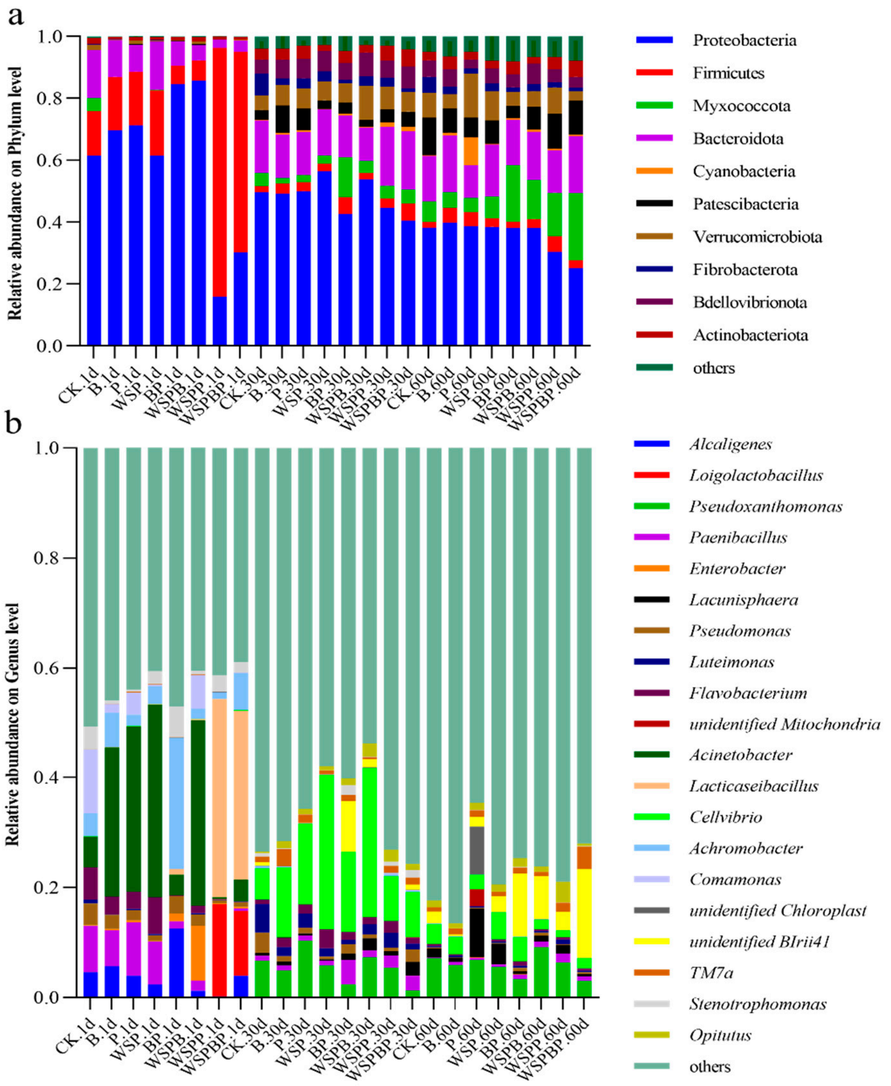

Figure S2 Fungal community composition at (a) phylum and (b) genus level during compost at day 1, 30 and 60, inoculated without (CK) or with *Bacillus subtilis* (B), *Paenibacillus* sp. (P), *Weissella paramesenteroides* (WSP) and their combinations (B+P, BP; WSP+B, WSPB; WSP+P, WSPP; and P+B+WSP, WSPBP) (relative abundance less than 1% is classified as others).

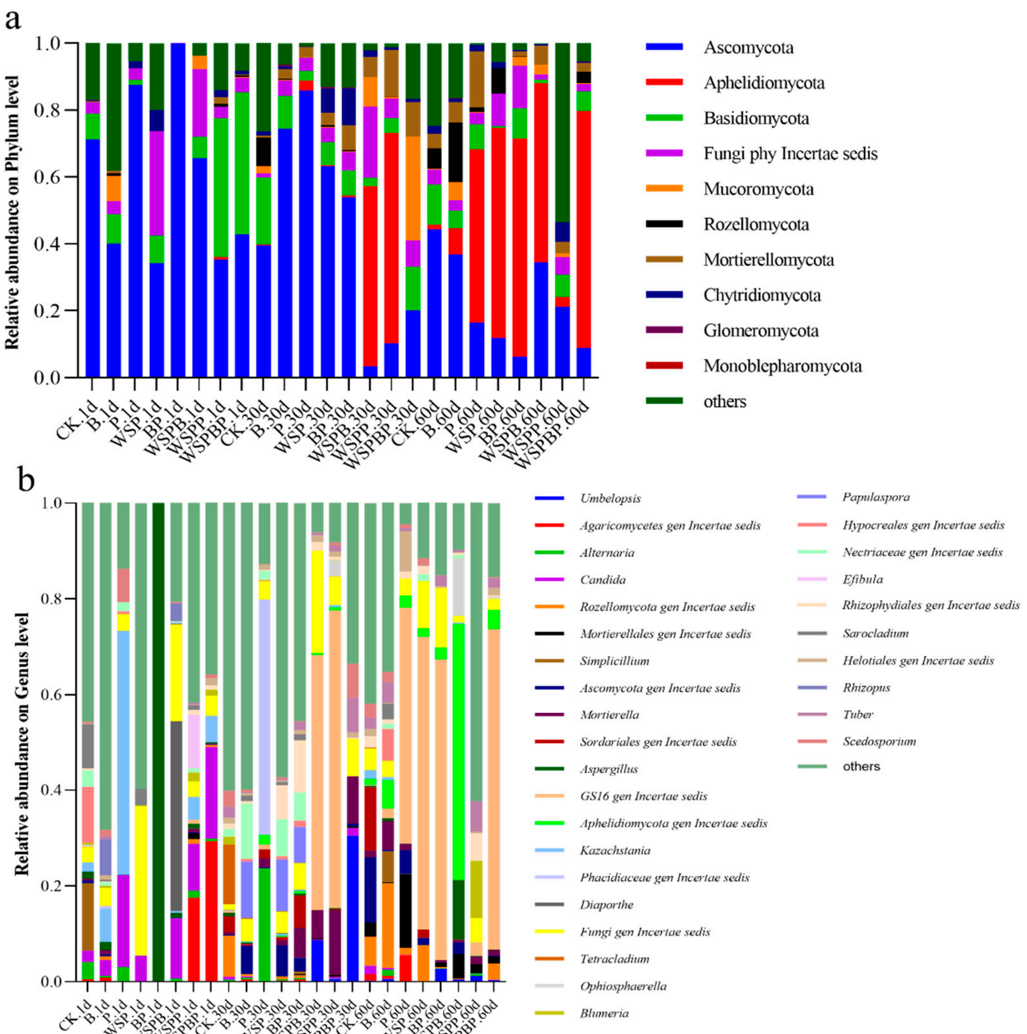

Figure S3 LEfSe analyses of the species with significant differences between groups in composting on day 1. Note: In the evolutionary branch diagram, the starting level is Phylum and the ending level is Species. Yellow nodes indicate no significant difference between the groups.

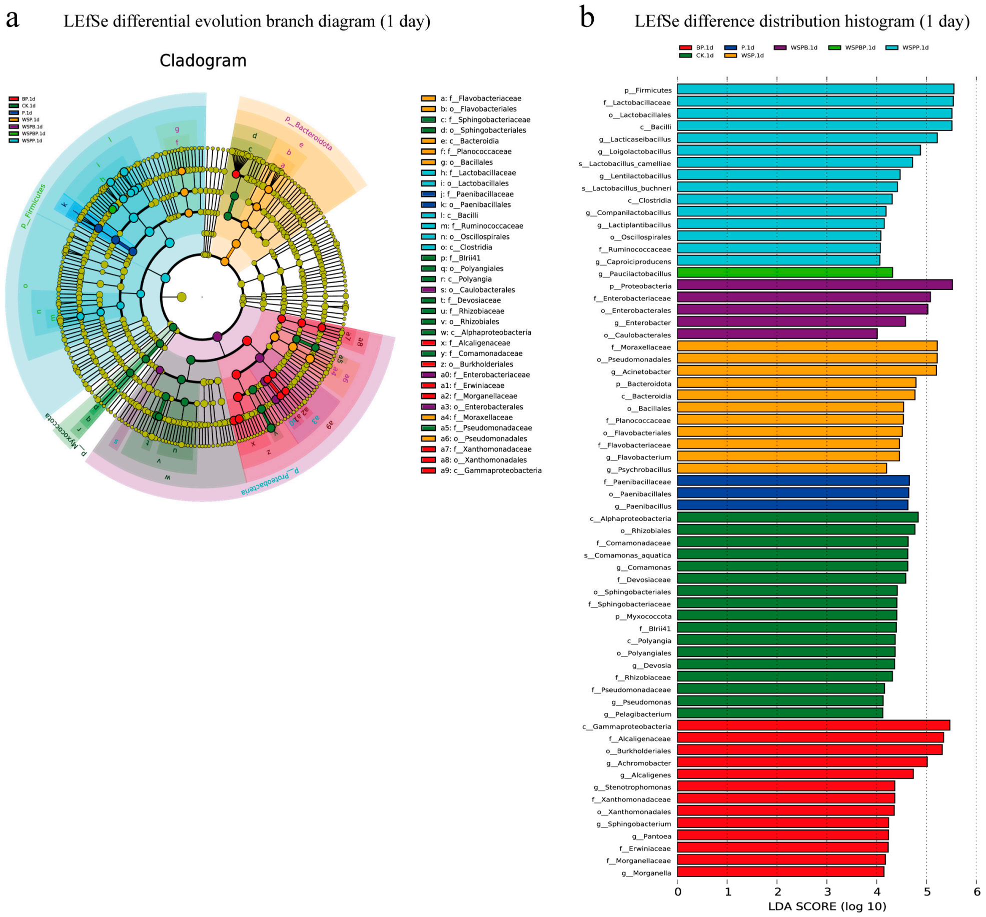

Figure S4 Redundancy analysis (RDA) of the correlation among the microbial communities and physicochemical parameters based on bacteria community (16S) and fungal community (ITS) at the genus level on day 30 and 60.

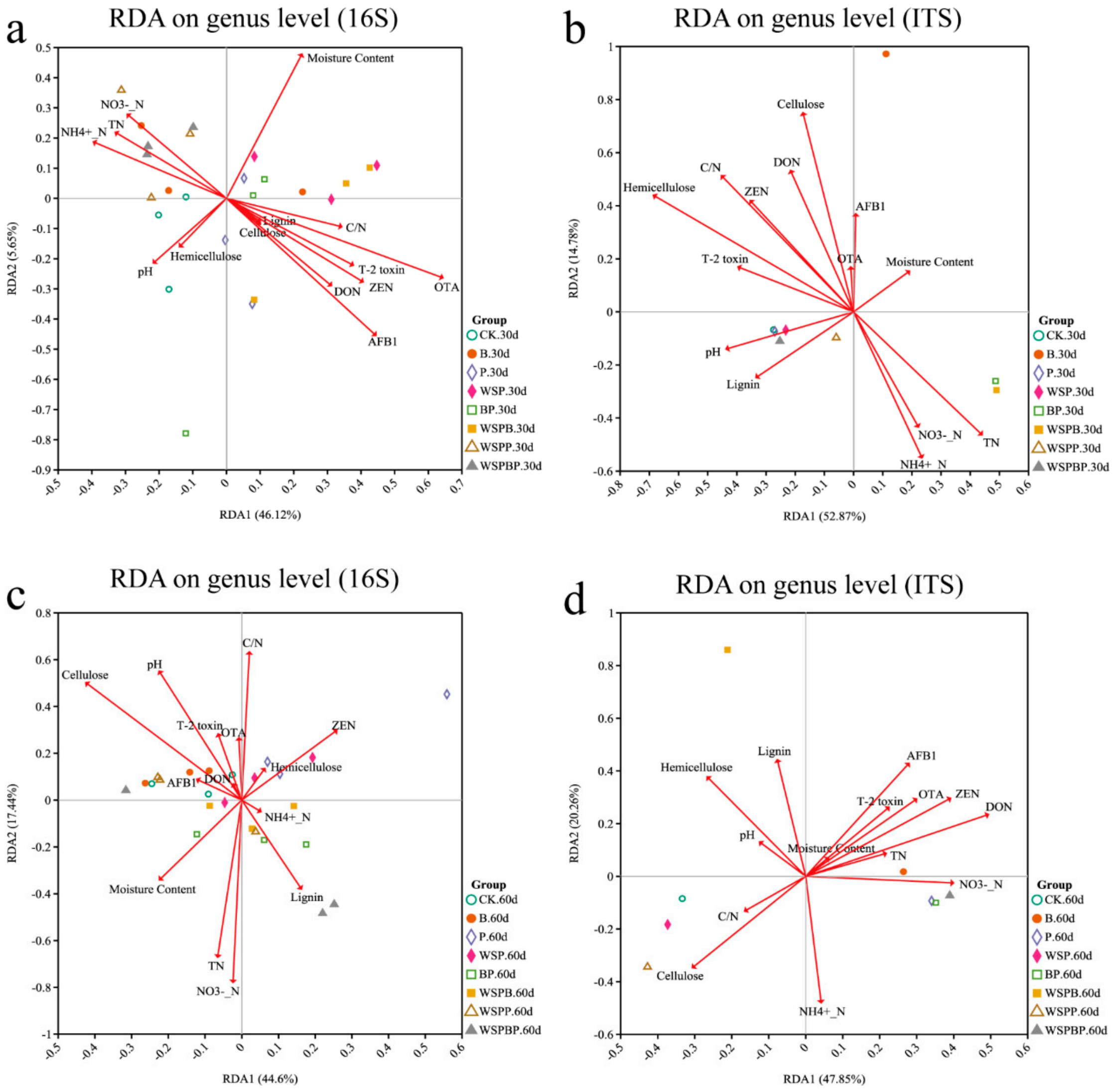

Table S1 Content results of mycotoxin changes in different treatments during compost. Different lowercase letters indicate significant differences between treatments (p<0.05). Different capital letters in-dicate significant differences between treatments (p<0.05). Equal letters indicate insignificant differences (p>0.05).

Items	Days	Treatment (T)								SEM	*P*-value		
		CK	B	P	WSP	BP	WSPB	WSPP	WSPBP		T	D	T×D
T2	30	4.75Abc	4.74Abc	6.08Aa	5.33Aab	4.58Abc	3.70Ac	4.33Abc	3.77Ac	0.222	0.031	<0.001	0.013
	60	1.88B	2.52B	1.88B	1.88B	1.88B	2.15B	1.56B	2.00B				
OTA	30	4.45Aab	5.05Aab	4.97Aab	5.59Aa	4.98Aab	4.55Aab	3.61Ab	4.60Aab	0.216	0.116	<0.001	0.667
	60	2.18B	2.70B	2.35B	2.40B	1.83B	2.58	1.73B	2.70B				
ZEN	30	4.83Aab	5.00Aab	5.17Aa	5.12Aab	3.80Aab	3.94Aab	4.04Aab	3.61Ab	0.178	0.015	<0.001	0.396
	60	2.36Bab	3.01Ba	2.89Ba	2.92Ba	2.03Bab	2.78Bab	1.68Bb	2.75Bab				
AFB1	30	4.10A	4.46A	4.11A	3.74A	3.43A	4.04A	3.13A	3.34A	0.189	0.384	<0.001	0.831
	60	1.52B	1.74B	1.55	1.35B	1.36B	1.86B	1.19B	1.87B				
DON	30	3.80Aab	4.20Aa	3.88Aab	4.10Aab	3.10Aabc	2.87Aacb	2.76Abc	2.43Ac	0.162	0.088	<0.001	0.027
	60	1.19B	1.92B	1.80B	1.39B	1.46B	1.76B	1.39B	2.03B				
